# Recommendations for antimicrobial stewardship during end-of-life patient care

**DOI:** 10.1017/ash.2022.98

**Published:** 2022-05-16

**Authors:** Erin Balkenende, Cassie Goedken, Daniel Livorsi, Karleen Giannitrapani, Matthew McCaa, Eli Perencevich

## Abstract

**Background:** Antimicrobials are frequently used during end-of-life care and may be prescribed without a clear clinical indication. Overuse of antimicrobials is a major public health concern because of the development of multidrug resistant organisms (MDROs). Antimicrobial stewardship programs are associated with reductions in antibiotic resistance and antibiotic-associated adverse events. We sought to identify and describe opportunities to successfully incorporate stewardship strategies into end-of-life care. **Methods:** We completed semistructured interviews with 15 healthcare providers at 2 VA medical centers, 1 inpatient setting and 1 long-term care setting. Interviews were conducted via telephone between November 2020 and June 2021 and covered topics related to antibiotic prescribing for hospice and palliative-care patients, including how to improve antimicrobial stewardship during the end-of-life period. We targeted healthcare providers who are involved in prescribing antibiotics during the end-of-life period, including hospitalists, infectious disease physicians, palliative care and hospice physicians, and pharmacists. All interviews were recorded, transcribed, and analyzed using consensus-based inductive and deductive coding. **Results:** End-of-life care, particularly hospice care, was described as an underutilized resource for patients, who are often enrolled in their final days of life rather than earlier in the dying process. Even at facilities with established antimicrobial stewardship programs, healthcare providers interviewed believed that opportunities for antimicrobial stewardship in the hospice and palliative care settings were missed. Recommendations for how stewardship should be incorporated in end-of-life care included receiving feedback on antimicrobial prescribing, increasing pharmacist involvement in prescribing decisions, and targeted education for providers on end-of-life care, including the value of shared decision making with patients around antibiotic use. **Conclusions:** Improved antibiotic prescribing during end-of-life care is critical in the effort to combat antimicrobial resistance. Healthcare providers discussed antimicrobial stewardship activities during end-of-life patient care as a potential avenue to improve appropriate antibiotic prescribing. Future research should evaluate the feasibility and effectiveness of incorporating these strategies into end-of-life patient care.

**Funding:** None

**Disclosures:** None

